# Convolutional Neural Network and Motor Current Signature Analysis during the Transient State for Detection of Broken Rotor Bars in Induction Motors

**DOI:** 10.3390/s20133721

**Published:** 2020-07-03

**Authors:** Martin Valtierra-Rodriguez, Jesus R. Rivera-Guillen, Jesus A. Basurto-Hurtado, J. Jesus De-Santiago-Perez, David Granados-Lieberman, Juan P. Amezquita-Sanchez

**Affiliations:** 1 ENAP-Research Group, CA-Sistemas Dinámicos, Facultad de Ingeniería, Universidad Autónoma de Querétaro (UAQ), Campus San Juan del Río, Río Moctezuma 249, Col. San Cayetano, San Juan del Río C.P. 76807, Qro., Mexico; martin.valtierra@enap-rg.org (M.V.-R.); jesus.rooney.rivera@uaq.mx (J.R.R.-G.); jesus.alberto.basurto@uaq.mx (J.A.B.-H.); sapjj@uaq.mx (J.J.D.-S.-P.); 2 ENAP-Research Group, CA-Fuentes Alternas y Calidad de la Energía Eléctrica, Departamento de Ingeniería Electromecánica, Tecnológico Nacional de Mexico, Instituto Tecnológico Superior de Irapuato (ITESI), Carr. Irapuato-Silao km 12.5, Colonia El Copal, Irapuato, Guanajuato C.P. 36821, Mexico; david.granados@enap-rg.org

**Keywords:** broken rotor bars, convolutional neural network, current signals, induction motor, motor current signature analysis (MCSA), short-time Fourier transform, transient state

## Abstract

Although induction motors (IMs) are robust and reliable electrical machines, they can suffer different faults due to usual operating conditions such as abrupt changes in the mechanical load, voltage, and current power quality problems, as well as due to extended operating conditions. In the literature, different faults have been investigated; however, the broken rotor bar has become one of the most studied faults since the IM can operate with apparent normality but the consequences can be catastrophic if the fault is not detected in low-severity stages. In this work, a methodology based on convolutional neural networks (CNNs) for automatic detection of broken rotor bars by considering different severity levels is proposed. To exploit the capabilities of CNNs to carry out automatic image classification, the short-time Fourier transform-based time–frequency plane and the motor current signature analysis (MCSA) approach for current signals in the transient state are first used. In the experimentation, four IM conditions were considered: half-broken rotor bar, one broken rotor bar, two broken rotor bars, and a healthy rotor. The results demonstrate the effectiveness of the proposal, achieving 100% of accuracy in the diagnosis task for all the study cases.

## 1. Introduction

IM is considered the most used electrical machine in industrial applications due to its features such as easy maintenance, great performance, low cost, and versatility [1]. Even though IM is a robust and reliable machine, it is susceptible to suffer diverse types of faults during its service life because of different thermal, electrical, and mechanical stresses produced during its operation [2,3]. Among the faults that can occur in IMs, e.g., broken rotor bars (a cracked bar), damaged bearings, unbalances, mixed eccentricities, and winding faults, among others, the broken rotor bar (BRB) (a fault produced by excessive temperature, dynamic forces, and high currents generated into the rotor cage) has become one of the most studied faults, since it allows the IM to operate with apparent normality; however, if the fault is not detected and corrected at stages of low severity, it can lead to the shutdown of processes and cause time and economical losses, as well as, in certain cases, putting at risk the operator and other machines connected to the same production line since it alters the consumed current and produces new frequency components [3,4]. To schedule maintenance times and avoid economic and human catastrophes, the development and application of diagnostic methods that offer more efficient and reliable results in terms of complexity and accuracy are still tasks of paramount importance, mainly considering BRB conditions at low severity, e.g., partially-broken rotor bars. In this regard, many diagnosis methods based on diverse physical magnitudes such as current, vibration, ultrasound, temperature, and magnetic flux, among others, have been employed for identifying the BRB fault, being MCSA the most preferable magnitude because it allows measuring the physical characteristics of an IM without interrupting its normal operation [5,6,7,8]. MCSA is employed for identifying the frequency components associated with specific faults; in particular, the MCSA attempts to identify the frequency components around the fundamental component (e.g., 50 or 60 Hz), which are related to the BRB fault [9]. In this sense, diverse works have focused on evaluating one or multiple BRBs, a consolidated fault (one or more bars completely segmented or cracked in two parts) [10,11,12]; however, few works have investigated a partially cracked bar, an initial condition of the BRB fault [9,13], because this condition alters slightly the monitored physical magnitudes, which increases the detection difficulty [14].

In the last decade, diverse machine learning-based methods have been introduced in the literature for BRB identification, where two main stages are carried out: (a) feature extraction; and (b) classification/pattern recognition [15]. In the feature extraction stage, the measured physical magnitudes of IMs are processed through different signal processing methods to obtain features or patterns that allow establishing a relationship with the IM condition. On the other hand, in the classification stage, the obtained features are employed for designing and training different pattern recognition algorithms, which automatically determine the IM condition [16]. In this regard, the fast Fourier transform [17,18], statistical methods [19,20], Welch method [21], regressive-based models [22], fractality-based method [23], entropy-based methods [24,25], multiple signal classification method [26], wavelet transform [27,28,29], empirical mode decomposition [30,31], and principal component analysis [32], among other indices or methods, have been explored to extract patterns about the IM condition. In a similar venue, different pattern recognition algorithms have already been presented to diagnose the IM condition automatically, e.g., artificial neural networks [4], fuzzy logic systems [23], k-means [33], support vector machines [34], and decision trees [35], among others. Notwithstanding the obtaining of promising results in the above-mentioned works, those techniques or algorithms present diverse issues that can compromise their performance in real-life situations, for instance: (1) a fine-tuning (a procedure performed typically by trial-and-error) of diverse parameters such as decomposition level, wavelet mother, model order, among others, for properly analyzing the in-test signals is required [36]; (2) noisy signals with nonstationary properties as the ones measured in the IMs degrades somehow their performance [37]; and (3) the adroit integration of feature (or set of features) and classifier is achieved by trial and error, where in all the cases the researcher proposes, tests, and selects the features to be used, which, on the one hand, increases the complexity and, on the other hand, might not lead to the best results [15]. From these points of view, the correct evaluation of the IM condition cannot be guaranteed [38]. As an alternative to lessen the limitations encountered in traditional machine learning-based methods, a new branch of machine learning named deep learning has been explored, where the CNN has become one of the most promising and widely used methods in several research fields [39], e.g., identification of cardiac rhythm problems in humans [40], health condition assessment of civil structures [41], object recognition [42], and identification of consolidated faults in rotatory machines (damaged bearings, stator winding faults, BRBs, and unbalanced rotor) [43,44,45,46,47], among other applications, outperforming the conventional machine learning methods in speed and accuracy [48]. In general, CNNs are presented as a single learning block that combines and performs both the feature extraction and the learning stage automatically and hierarchically [41]. The automatic feature extraction is one of the most important advantages of CNNs since it avoids the need to propose, extract, and test different features in order to assess which ones are the best-suited features [44]. This fact simplifies the design complexity and increases the classification effectiveness since somehow the human interpretation is canceled out. On the contrary, CNN implements in automatic way a set of filters to extract relevant features from input images [46]. In this sense, the short-time Fourier transform (STFT) and the wavelet transform have been used to transform time-series signals in time-frequency planes to be treated as input images which allow the CNN design [43]. In particular, the STFT is a low complexity time–frequency method capable of analyzing non-stationary signals; however, it can decrease its performance due to the leakage problem or the embedded noise in the current signals, which can compromise the design of an adequate CNN to evaluate the IM condition [49]. However, its low computational burden has attracted and motivated the development of improved STFT-based methods, i.e., the STFT technique followed by other methods [50].

The contribution of this work is the proposal of the adroit integration of a STFT-based method and the CNN to automatically identify and classify partially- and consolidated-BRB faults in IMs during the transient state. It is worth noting that the transient analysis is necessary for applications where the operating regimen varies continuously or in scenarios that require diagnosing the equipment before a prolonged activity time. In general, the proposal consists on the application of a notch filter to remove the fundamental frequency component of the current signal, the obtaining of its STFT-based time-frequency plane where the left sideband frequency component (LSFC) associated with the BRB fault is observed, and the CNN-based pattern recognition for automatic diagnosis. The proposed method was validated by using the experimental data of different IM conditions: a healthy (HLT) condition, half-BRB (HBRB) fault, and two consolidated BRB faults, one BRB (1BRB) and two BRBs (2BRBs). The obtained results show that the adroit integration of STFT and CNN methods is capable of identifying the healthy condition of IM and the presence of partially- and consolidated-BRBs with effectiveness of 100%.

## 2. Theoretical Background

### 2.1. Motor Current Signature Analysis

**Figure 1:**
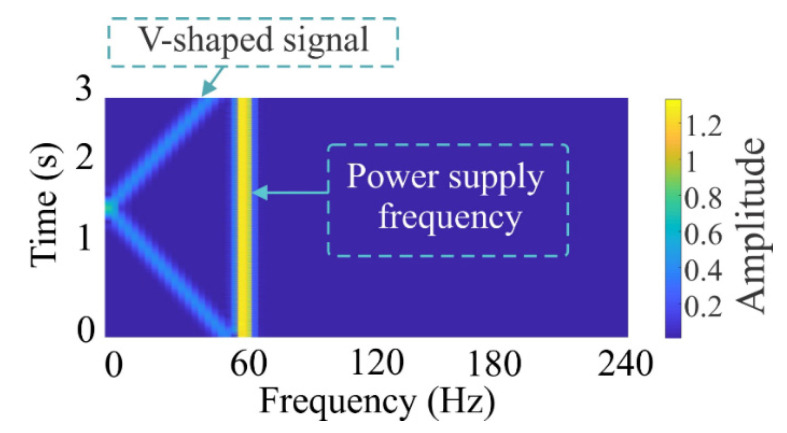
Time–frequency plane for an IM with a BRB condition by using the STFT and Equation (1) with a *fsupply* = 60 Hz and a time window of 3 s.

MCSA has become one of the most employed approaches for assessing the IM condition. It is used for identifying the frequency components contained in the measured current signals in order to associate them with a specific fault [50]. A BRB fault is characterized by producing sideband frequency components around the supply frequency (e.g., 50 or 60 Hz). In particular, the LSFC can be mathematically modeled by [51]:

(1) $$f_{\mathrm{LSFC}}=\left(1-2s\right)f_{\mathrm{supply}}$$

where *s* and *f*_supply_ represent the rotor slip and the power supply frequency, respectively. It should be pointed that, during the startup transient of an IM with a BRB condition, a V-shaped pattern is exhibited in a time–frequency plane due to the LSFC evolution (see Figure 1). In practice, the amplitude of this frequency component is affected by the fault severity and noise, being the partially-broken rotor bar the most difficult to detect since its amplitude varies slightly in comparison with the healthy condition [52]. Further, the power supply frequency limits the correct identification of the V-shaped frequency component because of the induced spectral leakage and its strong amplitude when the STFT technique is employed; hence, its elimination will allow observing with more clarity the V-shaped pattern [49].

### 2.2. Infinite Impulse Response (IIR) Notch Filter

Aiming to identify clearly the V-shaped frequency component associated with the motor condition, a digital IIR-based second-order notch filter, represented by Equation (2), is used to suppress out the main power supply frequency [53].

(2) $$F(z)=\frac{1-2\cos(\omega_{c}^{})z^{-1}+z^{-2}}{1-2r\cos(\omega_{c}^{})z^{-1}+r^{2}z^{-2}};\;\omega_{c}^{}=\frac{2\pi F_{c}}{F_{s}}$$

where *F_c_* and *F_s_* are the cutoff frequency (attenuated frequency) and the sampling frequency of the measured signal, respectively. *r* represents a factor that can only take values between 0 and 1. Let *x_k_* be the input of the filter in the actual sample *k* and *y_k_* the actual output; then, the digital notch filter can be implemented through the difference equation:

(3) $$y_{k}^{}=2r\cos\left(\omega_{c}^{}\right)y_{k-1}^{}-r^{2}y_{k-2}^{}+x_{k}^{}-2\cos\left(\omega_{c}^{}\right)x_{k-1}^{}+x_{k-2}^{}$$

where y*_k_*_−1_ and y*_k_*_−2_ are the past output samples of the filter and *x_k−_*_1_ and *x_k−_*_2_ are the past input samples.

The filter bandwidth, *BW*, is calculated as follows [54]:

(4) $$BW\approx \frac{F_{s}^{}\left(1-r\right)}{\pi }$$

**Figure 2:**
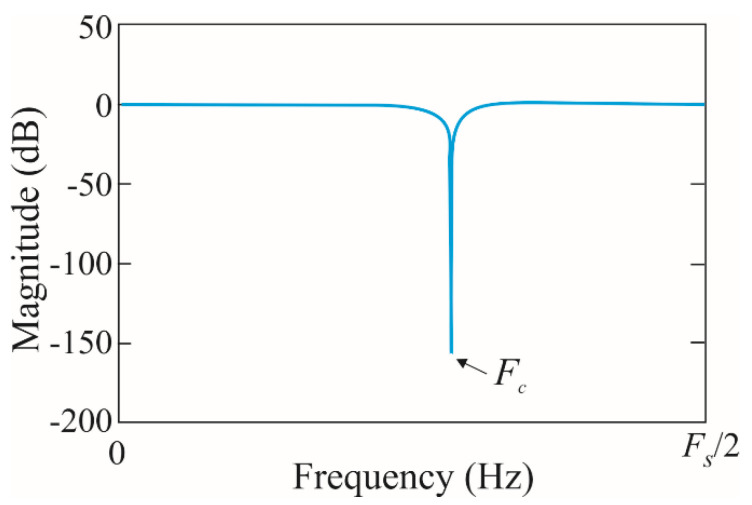
Frequency response of a notch filter with cutoff frequency *Fc* and a sampling frequency *Fs*.

*BW* depends on the r parameter, where a small r value leads to big filter bandwidth, whereas a value near 1 leads to a small *BW*. Figure 2 illustrates the frequency response of the IIR-based notch filter, *F_c_* must take the value of the main power supply frequency in order to enhance the V-shaped frequency component associated with the motor condition. It can be observed that the selected cutoff frequency will be eliminated satisfactorily without significantly affecting the amplitude of the remaining frequency components.

**Figure 3:**
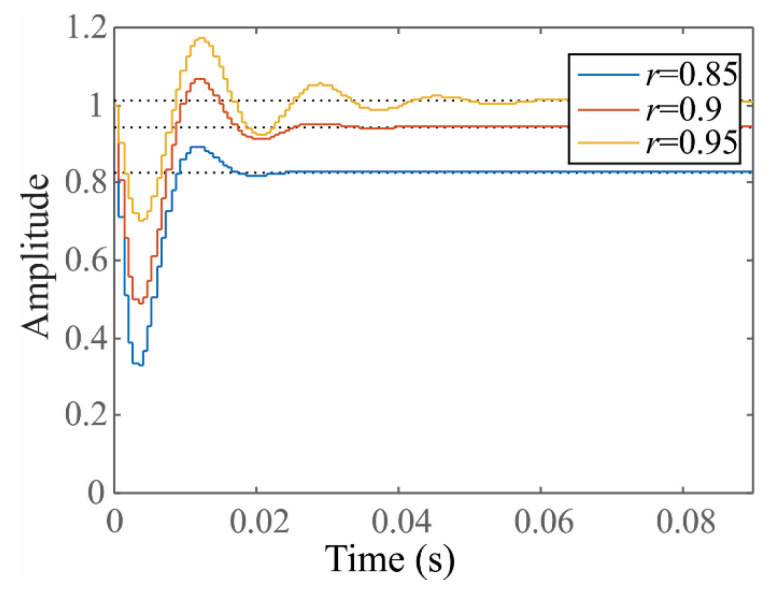
Notch filter response in the time domain for several *r* values.

The *r* factor must be selected carefully. A near-to-one value guarantees small bandwidth, but at expense of increasing the filter settling time [54]. Figure 3 depicts the filter response in the time domain for a step input and several *r* values close to one. Observing this figure, when *r* is 0.95, the response becomes slow; on the contrary, a *r* = 0.9 is more adequate since it converges faster than *r* = 0.95 and corresponds with a settling time ts of two cycles of the power supply frequency (ts ≈ 2/60 ≈ 0.033) [54]. Hence, the *r* value of 0.9 is used in this work.

### 2.3. Fourier Transform

**Figure 4:**
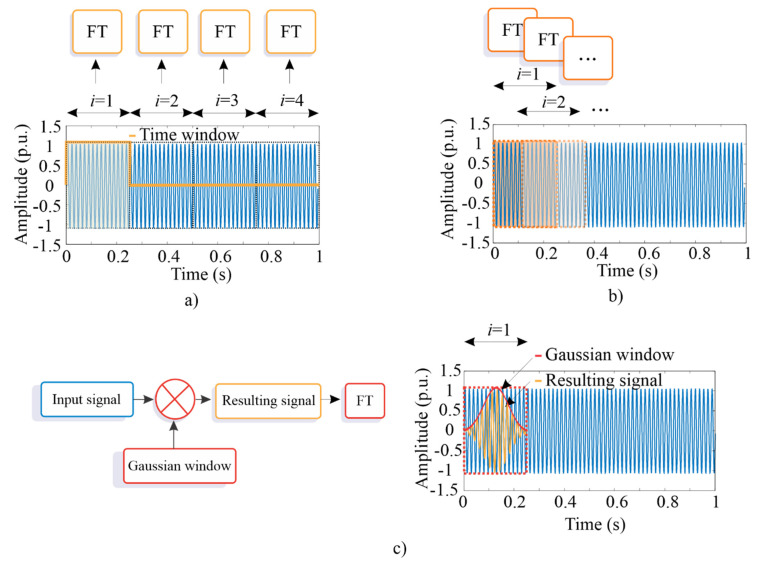
STFT schema: (**a**) without a time window overlapped; (**b**) with a time window overlapped; and (**c**) a time window (input signal) with a Gaussian window.

Once the supply frequency of time signals using a notch filter has been eliminated, the SFTF-based method is applied to obtain the time–frequency plane for the filtered signals, allowing the visualization of V-shaped patterns associated with the fault conditions. Fourier transform (FT) is a suitable method for identifying the frequency components of stationary signals [55]; however, its performance is degraded by analyzing noisy and non-stationary signals (signals with frequency components that vary over time), such as the measured ones in an IM during transient states [49]. To lessen this limitation, the STFT method, a variation of FT, is recommend for analyzing signals with non-stationary properties. In general, this method divides the original time-series signal into small time windows (see Figure 4a), where each segment is analyzed by means of the FT method, allowing observing the behavior of the frequency components over time [56]. It is important to mention that the selected time window defines the time and frequency resolution, i.e., longer time windows increase the frequency resolution but reduce the time resolution, and vice versa [57]. To reduce somehow this negative fact, the time windows can be overlapped, i.e., the next data segment only slides a percentage of the previous one (see Figure 4b). In addition, a window function (e.g., a Gaussian window) can be used to lessen the leakage problem as the product is zero-valued outside the window interval. After multiplication, the obtained signal is analyzed by the FT method (see Figure 4c) [58]. Therefore, the windowed STFT of a time-series signal *x*(*n*) is calculated by [57]:

(5) $$X(m,\omega )=\sum_{n=1}^{N}x[n]w[n-m]\;e^{-j\omega n}$$

where *w* is the window function centered at the sample *m*, *n* is a scalar index for the samples in the time signals, and *e*^−*jωn*^ represents the transformation kernel.

### 2.4. Convolutional Neural Network

**Figure 5:**
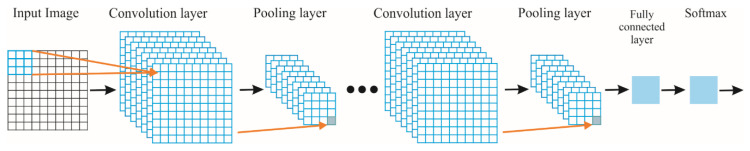
Convolutional Neural Network.

Finally, the images obtained by using STFT method are used to design a CNN for the diagnosis of an IM condition in an automatic way. CNN is a novel deep learning method used for pattern recognition in signals or images, which uses a single learning block to identify and classify in an automatic way the features in the input images and the desired outputs [59,60], avoiding hand engineering during the testing and selection of features. In general, the CNN is constituted by a network of multiple sub-CNNs which consists of a set of layers with one or more planes (see Figure 5).

According to Figure 5, the images are firstly set as inputs to the first sub-CNN known as convolution layer, which computes the dot product (convolutional operator), *, between the input image *X_i_*, with size *h* × *w*, and a set of convolutional filters *F_j_* to estimate certain features into the images. This operation is computed as follows [60]:

(6) $$y_{j}=\sigma \left(\sum F_{j}*X_{i}+B_{j}\right)$$

where *B* and *σ*(·) indicate a bias term and the nonlinear activation function, respectively. In particular, each *F_j_* of size *k*_1_ × *k*_2_ convolves with a local region of the input signal with stride *s*_1_ and shares the same weights. The resulting output, *Y_j_*, for each *F_j_*, known as feature maps, has a size of *z*_1_ × *z_2_*, which is determined as follows [61]:

(7) $$z_{1}=\frac{h-k_{1}+2p}{s_{1}}+1$$

(8) $$z_{2}=\frac{w-k_{2}+2p}{s_{1}}+1$$

where *p* is the zero-padding parameter. A value of 1 is recommended because the input and output spatial resolution must be the same [61]. There are diverse nonlinear activation functions such as sigmoid, hyperbolic tangent, rectified linear unit (ReLu), among others, being the ReLu, *f*(*Y_j_*) = max(0,*Y_j_*), the fastest and most effective to learn the nonlinear properties of each feature map, *Y_j_*, in a CNN [62].

Then, the obtained feature maps, *Y_j_*, in the previous layer are used as input for other subsequent sub-CNN layers named pooling layers, which are employed for subsampling or contracting the dimensionality or resolution of feature maps with the aim of reducing the quantity of information to be processed, but retaining the relevant features determined in the previous sub-CNN [47]. It moves a filter of size *K*_1_ × *K*_2_ with a stride *s*_2_ across the feature maps by taking the average (average pooling) or maximum (max pooling) of the neighbor values chosen by the filter. Hence, a sub-sampled representation of *Y_j_*, with a size of *Z*_1_ × *Z*_2_, is obtained as follows [62]:

(9) $$Z_{1}=\frac{z_{1}-K_{1}}{s_{2}}+1$$

(10) $$Z_{2}=\frac{z_{2}-k_{2}}{s_{2}}+1$$

It is important to mention that max pooling has presented better results than average pooling since it can capture invariant features correctly and improve the generalization performance [63]. For these reasons, the max pooling is employed in this work. In the last layer, all the feature map elements are connected to the fully connected layer, which is a standard neural network, i.e., a multilayer perceptron network, in order to perform pattern recognition. Finally, the softmax layer applies the softmax transfer function for generating the desired outputs. In this work, this layer determines the induction motor condition. A detailed explanation for CNNs can be found in [61].

## 3. Proposed Methodology

**Figure 6:**
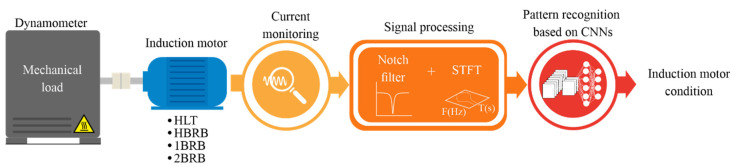
Proposed methodology.

Figure 6 shows the proposed methodology to detect BRBs in IMs. In general, it consists of three steps: current monitoring, signal processing, and automatic pattern recognition based on CNNs. In the first step, the current signal is acquired during the IM startup transient, where four rotor conditions, HLT, HBRB, 1BRB, and 2BRB, are considered. A brake dynamometer is used to provide mechanical load. In the signal processing step, two processing stages, a notch filter and the STFT method, are applied consecutively. The notch filter is applied to the signal to delete the strong energy of the fundamental frequency component and, thus, highlight the frequency components associated with the fault. Then, the STFT using both overlap and a Gaussian window is used to obtain the time–frequency plane of the current signal, allowing the visualization of V-shaped patterns associated with the fault conditions. Finally, in the pattern recognition step, a CNN is proposed to classify the IM condition in an automatic way. It is worth noting that the time–frequency plane obtained through the STFT is treated as an image in order to implement a conventional two-dimensional (2D) CNN. In the 2D CNN design, different image sizes, learning rates, and batch sizes are analyzed. The experimentation and the results are presented in the next section.

## 4. Experimentation and Results

### 4.1. Experimental Setup

**Figure 7:**
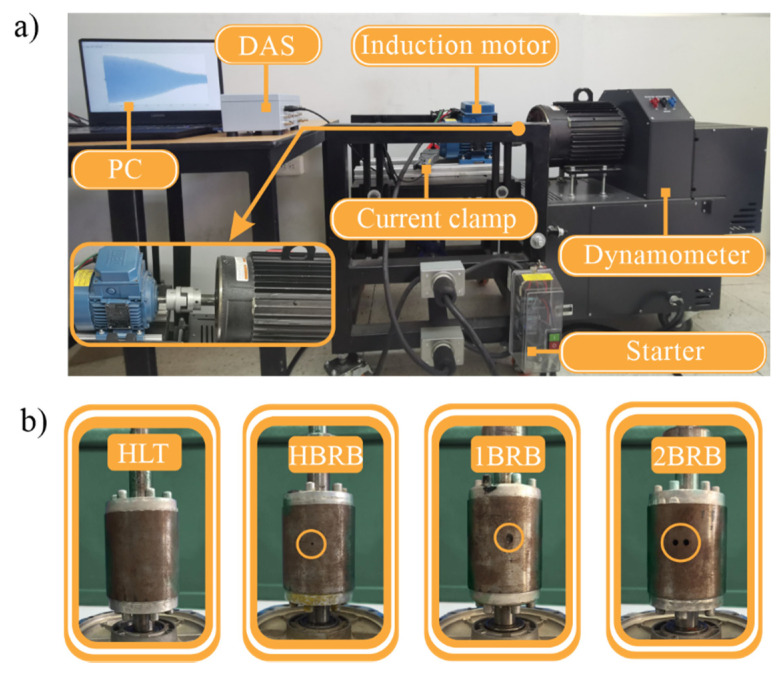
(**a**) Experimental setup; and (**b**) rotor conditions.

**Figure 8:**
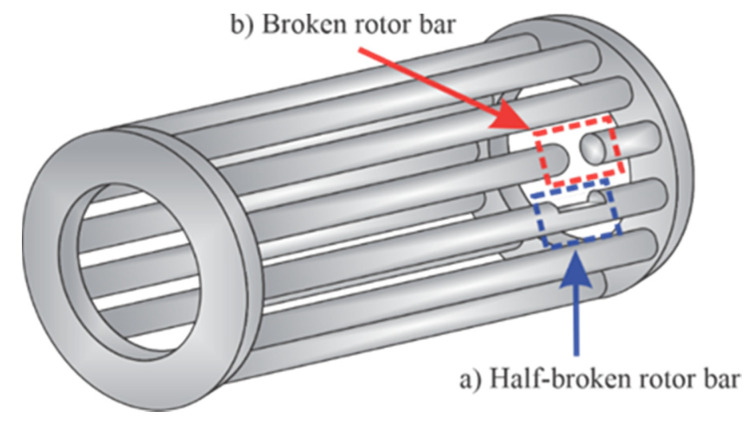
Crack deep for: (**a**) HBRB condition; and (**b**) BRB condition.

**Figure 9:**
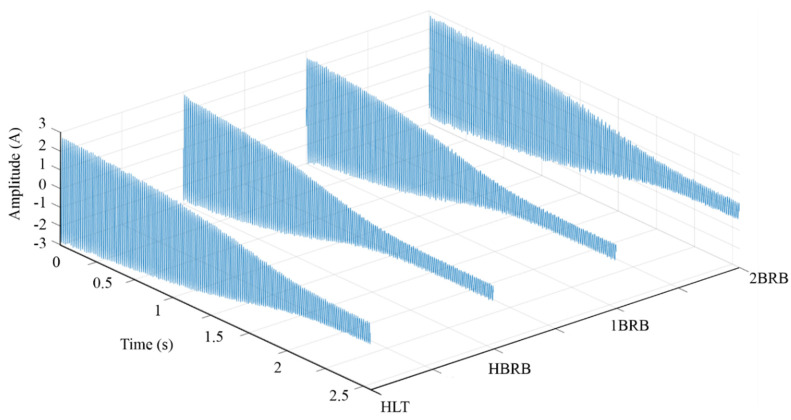
Current signals during the startup transient for HLT, HBRB, 1BRB, and 2BRB conditions.

The experimental setup used to validate the proposed methodology is shown in Figure 7a. The in-test motor (model WEG-00136APE48T) has two poles, 28 bars, nominal power of 1 hp, and is fed with 220 Vac at 60 Hz. A four-quadrant dynamometer (model 8540) from Lab-Volt is used to provide the mechanical load. Figure 7b shows the rotor conditions, i.e., HLT, HBRB, 1BRB, and 2BRB, where the fault conditions are artificially generated by following the next steps: (1) identify the bars into the rotor by means of an armature growler tester; and (2) use a computerized numeric control (CNC) machine to drill and broke the bar. The CNC machine was used to guarantee the accuracy for generating the partially-BRB and the consolidated BRBs. In particular, to generate a HBRB, a hole of diameter 2.10 mm with a depth of 5 mm is produced in a bar of the rotor. On the other hand, to generate a 1BRB and 2BRB, one or two holes with a depth of 10 mm in the squirrel cage was made, respectively. Figure 8 shows the crack deep for HBRB and BRB conditions, respectively. For the current signal acquisition, a current clamp model i200s from Fluke was used as a sensor, and then a data acquisition system (DAS) based on the NI-USB 6211 board from National Instruments, which was configured with a sampling frequency of 1500 samples/s and a time acquisition of 2.5 s, was used. These values allow capturing both the startup transient and the V-shaped pattern with enough time–frequency resolution, as shown in the next subsection [54]. The direct online starter method was used to start the IM. For each IM condition, 100 current signals were acquired in an automatic way by using solid-state relays. Figure 9 shows one of the acquired current signals for each IM condition. The overall methodology was implemented in a portable personal computer (PC) using MATLAB software.

It is important to mention that the experimental setup presented in this work was carried out to evaluate the IM condition when it is exposed to BRB faults since the benchmark studies or publications about the fault studied in this work are not found in the literature, unlike other studied faults such as bearings, where the proposed methodologies use mainly the databases and experimental setups provided by the Case Western Reserve University and the University of Cincinnati’s Center for Intelligent Maintenance Systems for comparing their results with other works [64,65,66,67,68].

### 4.2. Signal Processing Results

**Figure 10:**
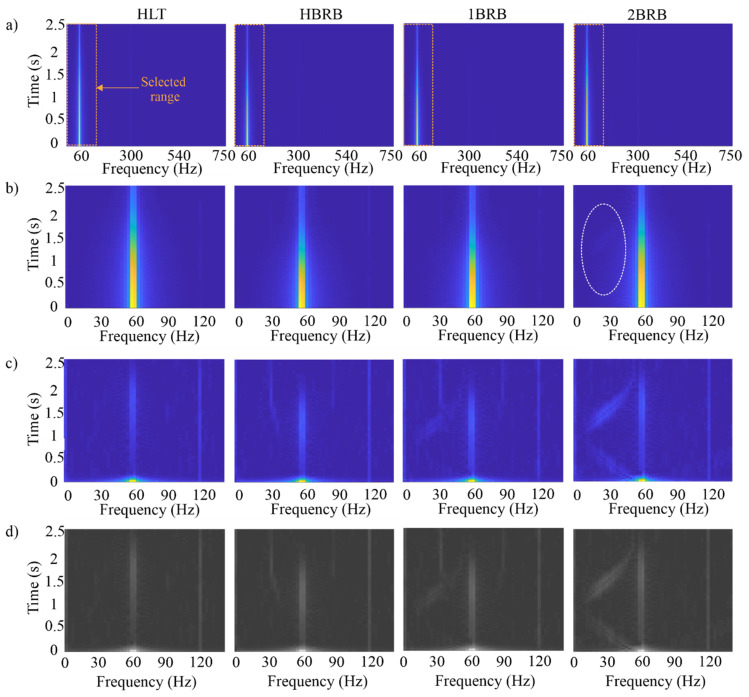
Time–frequency plane for: (**a**) the STFT of the current signals shown in Figure 9; (**b**) the STFT in the selected range; (**c**) the STFT after the Notch filter application in the time domain; and (**d**) the STFT in the selected range in grayscale.

**Figure 11:**
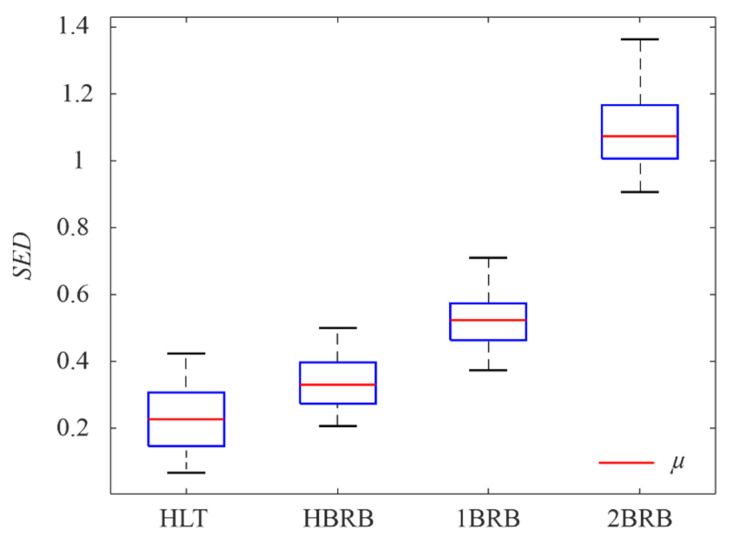
Boxplot for the *SED* values.

Once the current signals were acquired, the signal processing steps, the notch filter, and the STFT shown in Figure 6, were applied. To observe the advantages of applying the notch filter, the time–frequency results for the current signals are shown in Figure 10. Firstly, Figure 10a shows the results obtained through the STFT with overlap and Gaussian window in the available bandwidth, sampling frequency/2 = 1500/2 = 750 Hz. The analyzed time windows by the STFT comprehend 500 samples and an overlap of 10 samples. As the region of interest is smaller, only the range where the V-shaped pattern is located, 0 to 120 Hz, is selected (see Figure 10b). Although the application of the overlap and the Gaussian window in the STFT allow improving the time resolution for the evolution of the frequency components and reduce the leakage effect, the V-shaped pattern is not visible enough due to the strong influence of the fundamental frequency component; in fact, only the pattern in the 2BRB condition is barely noticeable (see Figure 10b, white dotted ellipse). However, when the fundamental frequency component is removed by means of the notch filter, the patterns associated with the BRB condition are more evident, as shown in Figure 10c. As can be observed, the proposal presents a suitable detectability since the frequency components associated with the BRB condition are detected even for the partially-broken rotor bar condition. To quantify the detectability of the V-shaped patterns, the spectral energy density (*SED*) for the time-frequency planes is presented. *SED* is computed as follows:

(11) $$SED={\left|X(f)\right|}^{2}$$

where *X*(*f*) is the Fourier transform (FT) of an input signal. In the STFT, the *SED* for each FT is accumulated. Figure 11 shows as boxplots the obtained results for all the tests in each condition by considering their mean (*μ*) and standard deviation (*σ*). As can be observed, the *SED* increases according to the fault severity, indicating that the obtained time–frequency planes provide sensitive information to the fault severity. If the means are normalized with respect to the healthy condition, the following values are obtained: *μ_HLT_/μ_HLT_* = 1, *μ_HBRB_/μ_HLT_* = 1.4832, *μ_1BRB_/μ_HLT_* = 2.3130, and *μ_2BRB_/μ_HLT_* = 4.8086. These values indicate the detection capacity in terms of *SED*, e.g., the proposal detects an increment of 48.32% in the *SED* for the HBRB condition by taking as reference the *SED* of the HLT condition. The overlap issues presented in Figure 11 are addressed by the CNN-based pattern recognition stage. Therefore, these time–frequency planes are treated as images in order to be the inputs for the 2D CNN; however, they are first converted to grayscale, as shown in Figure 10d, to reduce the complexity of the input image; a 3D pixel value (Red, Green, and Blue) is converted to a 1D value (Gray), without affecting the observed pattern. The CNN configuration parameters and its results are presented in the next subsection.

### 4.3. Convolutional Neural Network Results

**Figure 12:**
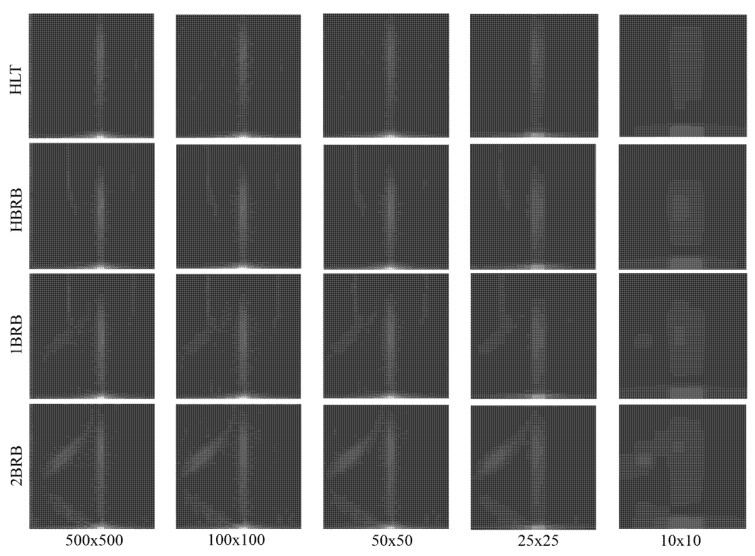
Images for different sizes.

As the input image size is fundamental in the CNN complexity, a tradeoff between the information quantity that can be extracted from the analyzed image and the image size has to be established. Figure 12 shows the obtained results for five different sizes, i.e., 500 × 500 (original size), 100 × 100, 50 × 50, 25 × 25, and 10 × 10 pixels. From a visual inspection, the images with a size of 25 × 25 pixels were selected as inputs for the 2D-CNN since they keep the information that is observed in larger images but with a lower computation cost because the matrix size is reduced. It is worth noting that the image size can be optimized by means of multi-objective optimization algorithms; however, the used value is somehow suitable by considering that other CNN-based approaches use input images with sizes of 224 × 224 [43].

**Figure 13:**
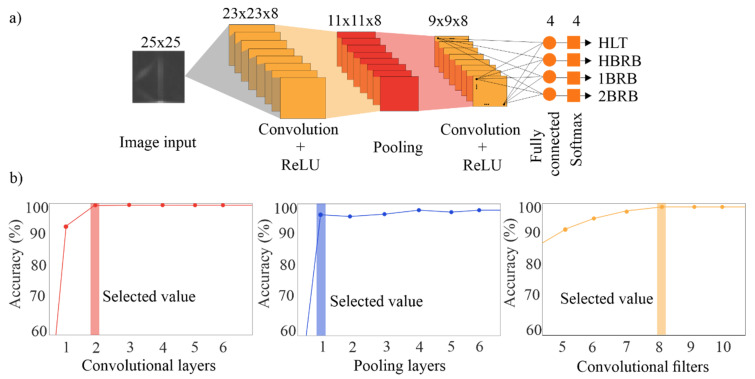
(**a**) Resulting convolutional neural network (CNN) architecture; and (**b**) accuracy results for different convolutional layers, pooling layers, and convolutional filters.

Once the input image size is defined, the CNN architecture can be constructed. After testing different numbers of convolutional layers, convolutional filters, and pooling stages by means of trial and error, the highest effectiveness with the simplest architecture was obtained for the architecture shown in Figure 13a. Figure 13b shows the accuracy results for the different trial and error scenarios carried out in the above-mentioned tests. It is worth noting that the parameters were changed one at a time. The selected values in Figure 13b are the lowest values with the highest accuracy. Therefore, the CNN consists of two convolutional layers with eight sliding convolutional filters and rectified linear unit (ReLU) layers, one max pooling layer, one fully connected layer, and one softmax layer. A second pooling layer was not required due to the small size of the last feature maps, i.e., 9 × 9. The fully connected layer size is equal to the number of classes in the target data, four (HLT, HBRB, 1BRB, and 2BRB). These parameters are summarized in Table 1. Although promising results were obtained, a strict, systematic, and multi-objective optimization procedure for the entire CNN architecture is still needed.

**Figure 14:**
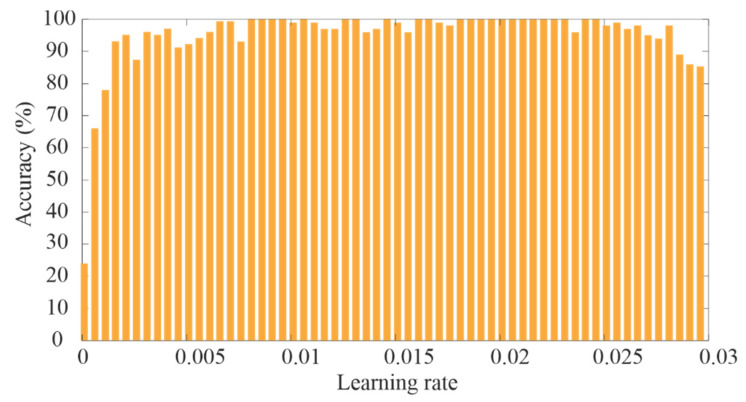
Obtained accuracy for different learning rate values.

**Figure 15:**
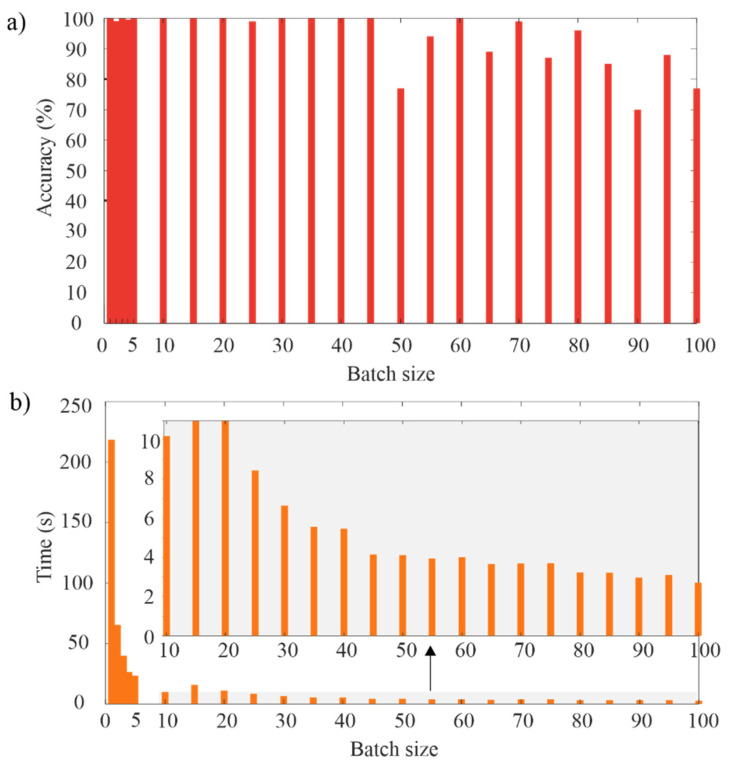
Results for different batch size values: (**a**) accuracy; and (**b**) computational time.

Once the general CNN architecture has been defined, a finer selection of other parameters such as learning rate and batch size can be carried out. The learning rate determines the step size to adjust the weights and reduce the error during the training. Figure 14 shows the obtained results for different learning rates by considering only one epoch. One epoch is a complete pass through the entire dataset. As can be observed, the extreme values compromise negatively the accuracy; therefore, in this work, a learning rate value of 0.02 was used since it presents a high accuracy and can accelerate the error convergence. On the other hand, Figure 15 shows the obtained results for the accuracy and computational time using different values of batch size. The batch size determines the size of a subset of the entire dataset that is used in each training iteration. As can be observed in Figure 15, a small value of batch size generates a high accuracy but a high computational time; on the contrary, a high value of batch size reduces the computational time but the accuracy is negatively compromised. In this regard, a batch size of 30 was selected since it offers high accuracy and a suitable computational time.

**Figure 16:**
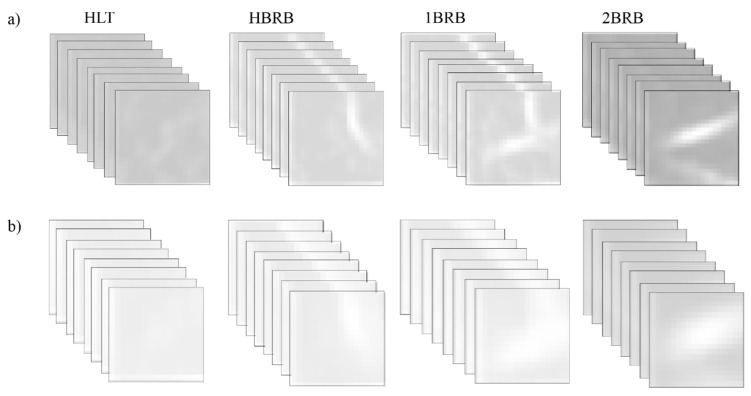
Feature maps for: (**a**) the first convolutional layer; and (**b**) the second convolutional layer.

**Figure 17:**
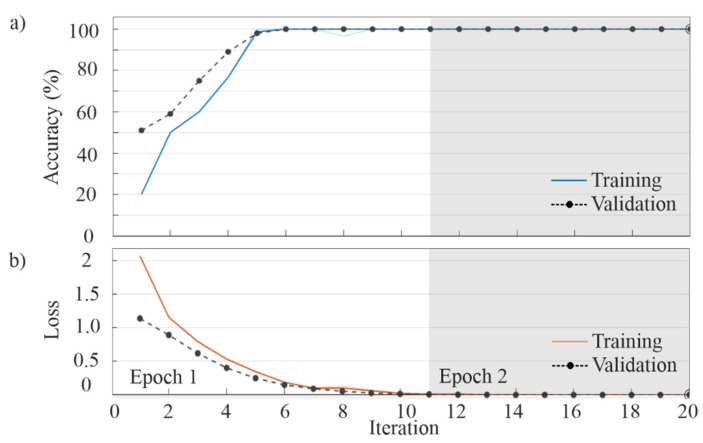
CNN training and validation: (**a**) Accuracy and (**b**) Loss.

After the selection of the above-mentioned parameters, the CNN can be completely trained and validated. From the entire dataset (400 current signals, i.e., 100 of each IM condition), 75% (300 current signals) was used for training and the remaining 25% (100 current signals) for validation. In this work, the stochastic gradient descent with momentum optimizer was used as the training algorithm [69]. Figure 16a,b shows the extracted patterns by the CNN for each IM condition in the first and second convolutional layers. As can be observed, these patterns correspond to the V-shaped pattern associated with the BRB condition. It is worth noting that they are automatically extracted and considered as features by the CNN. Figure 17 shows the obtained results for accuracy and loss, where it is observed that an accuracy of 100% is obtained during the first epoch for both training and validation datasets. Table 2 corroborates the obtained accuracy since a perfect match is observed between the target class and the predicted class (confusion matrix), demonstrating the proposal effectiveness with 100% for all the study cases.

### 4.4. Comparison with Previous Works

Table 3 summarizes the results obtained by using the proposed methodology and previous works recently reported in the literature, where the methods employed, the evaluated damage level, and the obtained effectiveness percentage are presented. According to Table 3, the proposed method presents effectiveness of 100% for detecting a partially-BRB fault as well as the consolidated state (1BRB and 2BRB), unlike other methods presented in the literature [10,12,43], which are focused mainly on evaluating IMs with one or more BRBs. In particular, promising results were also obtained using pre-trained CNNs such as the VGG-16 architecture [43]; however, although the design is easy, it keeps the complexity of a CNN for general applications, which in some cases is neither necessary nor justified, mainly if the task is not a large-scale image recognition problem. On the other hand, it is worth noting that in many works the in-test fault severity is associated with the detection capacity of the used signal processing techniques since the higher is the severity, the easier is the detection; for instance, the CWT is used to detect three BRBs in [43], whereas the STFT is used in this work to detect HBRB, showing the usefulness of STFT for the analysis of current signals in transient state.

Low fault severities of BRB, e.g., partially-BRB, are characterized by producing imperceptible alterations or changes into the measured signals in comparison with the signals of a healthy IM, making its detection a challenging task. However, this condition has been considered by diverse works in the literature [9,23,26,70,71], reaching an accuracy higher than 95%. Despite obtaining promising results, the testing and assessment of multiple indices or features to work with the proposed classifier are hand-engineered, which, on the one hand, increases the complexity and, on the other hand, might not lead to the best possible results. On the contrary, the proposed CNN-based methodology consists of a single learning block for automatically determining and classifying the features found into the images, making it a more attractive tool for the developer since exhaustive testing and selection of features based on linear and nonlinear indices to properly evaluate the IM condition, even for small frequency changes associated with partially-broken rotor bars, are not required.

## 5. Conclusions

Fault detection in IMs is of paramount importance for the industry. In this work, a methodology based on the notch filter, STFT, and CNN is proposed to detect broken rotor bars in IMs from partially-BRBs (i.e., HBRB) to consolidated-BRBs (i.e., 1BRB or 2BRB). Firstly, the notch filter application allows removing the fundamental frequency component of the current signal during the startup transient, thus highlighting or making more evident the information associated with the fault condition. Then, the STFT featuring overlap and Gaussian window are applied to obtain the V-shaped pattern in the time-frequency plane, improving the time resolution and reducing the leakage effect. Finally, the obtained time-frequency planes are treated as images and inputs to the 2D CNN in order to carry out the automatic fault detection. In the CNN design, several configuration parameters were tested, i.e., different values for the input image size, learning rate, and batch size. After the tests, an input size of 25 × 25, a learning rate of 0.02, and a batch size of 30 were selected according to a tradeoff between accuracy and computational time; although exhaustive experimentation was carried out, the application of optimization algorithms is open for the CNN architecture improvement, including the image/input size.

As study cases, four IM conditions were considered, HBRB, 1BRB, 2BRB, and HLT, where classification effectiveness of 100% was achieved in all study cases, demonstrating the potential of the proposal for fault diagnosis. It is important to mention that the proposed method can be a suitable tool to identify the IM condition into industrial processes since it only requires monitoring the IM current to diagnose in an automatic way the BRB fault without interrupting its normal operation. In a future work, other faults and their individual MCSA-based diagnosis schemes will be investigated to integrate and develop a more general CNN-based diagnosis system through incremental training. In addition, the study of incipient faults by using accelerated degradation test platforms will be conducted.

## Figures and Tables

**Table 1:** CNN configuration.

Name	Type	Activations	Learnables
Input	Image input	25 × 25 × 1	-
Conv_1	Convolution	23 × 23 × 8	Weights 3 × 3 × 1 × 8 and Bias 1 × 1 × 8
Relu1	Rectified linear unit	23 × 23 × 8	-
2 × 2-MP	Max pooling	11 × 11 × 8	-
Conv_2	Convolution	9 × 9 × 8	Weights 3 × 3 × 8 × 8 and Bias 1 × 1 × 8
Relu2	Rectified linear unit	9 × 9 × 8	-
FC	Fully connected	1 × 1 × 4	Weights 4 × 648 and Bias 4 × 1
SM	Softmax	1 × 1 × 4	-
Class	Classification output	-	-

**Table 2:** Confusion matrix.

			Target Class		
**Predicted class**		**HLT**	**HBRB**	**1BRB**	**2BRB**
	**HLT**	25	0	0	0
	**HBRB**	0	25	0	0
	**1BRB**	0	0	25	0
	**2BRB**	0	0	0	25
	**Total accuracy (%)**				**100**

**Table 3:** Results and characteristics offered by the proposed work and previous methods.

Work	Proposed Methods	Damage Level	Accuracy (%)
[9]	1. Feature extraction is performed by using Homogeneity analysis 2. Gaussian probability density function is employed as classifier.	HBRB, 1- and 2BRB	99
[10]	1. Features extraction is performed by using MUSIC technique 2. Bayes method is employed as classifier.	1- and 2BRB	100
[12]	1. Features extraction is performed by using Wavelet and Hilbert transforms. 2. Linear discriminant technique is employed as classifier.	1- and 2BRB	100
[23]	1. Feature extraction is performed by using Fractal dimension 2. Fuzzy logic is employed as classifier.	HBRB, 1- and 2BRB	95
[26]	1. Features extraction is performed by using extended Kalman filter 2. MUSIC technique is employed as classifier.	HBRB and 1BRB	100
[43]	1. Wavelet transform is used to transform the measured signals to images. 2. A CNN is employed as features estimator and classifier.	3BRB	99
[70]	1. Features extraction is performed by using Wavelet transform. 2. Correlation Pearson is employed as classifier.	HBRB, 1- and 2BRB	95
[71]	1. Feature extraction is performed by using Hilbert transform. 2. Gaussian probability density function is employed as classifier.	HBRB, 1- and 1½BRB	99
Proposed work	1. Short time Fourier transform is used to transform the measured signals to images. 2. A CNN is employed as features estimator and classifier.	HBRB, 1- and 2BRB	100
